# Enhanced transoral outlet reduction using endoscopic submucosal dissection and zipper-type suture technique after Roux-en-Y gastric bypass

**DOI:** 10.1016/j.vgie.2025.06.005

**Published:** 2025-06-25

**Authors:** Davekaran Buttar, Mayank Goyal, Ashwariya Ohri, Barham K. Abu Dayyeh

**Affiliations:** 1 Division of Gastroenterology and Hepatology, Mayo Clinic, Rochester, Minnesota, USA; 2 Division of Gastroenterology and Hepatology, Department of Internal Medicine, Cedars-Sinai, Los Angeles, California, USA

## Abstract

**Background and Aims:**

Weight regain after Roux-en-Y gastric bypass is common and often linked to anatomical changes such as gastrojejunal anastomosis (GJA) dilation and pouch enlargement. Transoral outlet reduction (TORe) offers a minimally invasive alternative to surgical revision. We present a modified TORe technique incorporating submucosal dissection, argon plasma coagulation, zipper-style GJA suturing, and distal pouch tubularization.

**Methods:**

A 53-year-old woman with post-Roux-en-Y gastric bypass weight recurrence and GERD underwent enhanced TORe. The procedure included submucosal dissection of the GJA, argon plasma coagulation, zipper-pattern endoscopic suturing for GJA reduction, and tubularization of the distal gastric pouch.

**Results:**

At 6-month follow-up, the patient achieved 14% total body weight loss and resolution of GERD symptoms. No adverse events occurred.

**Conclusions:**

This modified zipper-TORe approach is safe and efficient and may enhance durability and clinical outcomes.

## Introduction

Weight recurrence is common after bariatric surgeries. Patient factors include medical, genetic, dietary, psychological, and behavioral components, whereas anatomical factors include development of a gastrogastric fistula, dilation of gastrojejunal anastomosis (GJA), and enlargement of the gastric pouch. Surgical revision and conversion of bariatric surgery are effective; however, they are technically challenging and are associated with morbidity compared with an index procedure. Therefore, endoscopic intervention has emerged as a minimally invasive, safe, and effective treatment option.^1^ Transoral outlet reduction (TORe) is an effective technique for managing weight gain after Roux-en-Y gastric bypass (RYGB) by reducing the diameter of the GJA and size of the gastric pouch. We developed a modified approach to TORe. These modifications include endoscopic submucosal dissection with argon plasma coagulation, zipper-type suture reduction of the GJA, and distal tubularization of the gastric pouch. With these modifications, we aim to streamline the learning curve for practitioners while potentially offering more-robust long-term outcomes for patients.

### Case presentation

**Figure 1:**
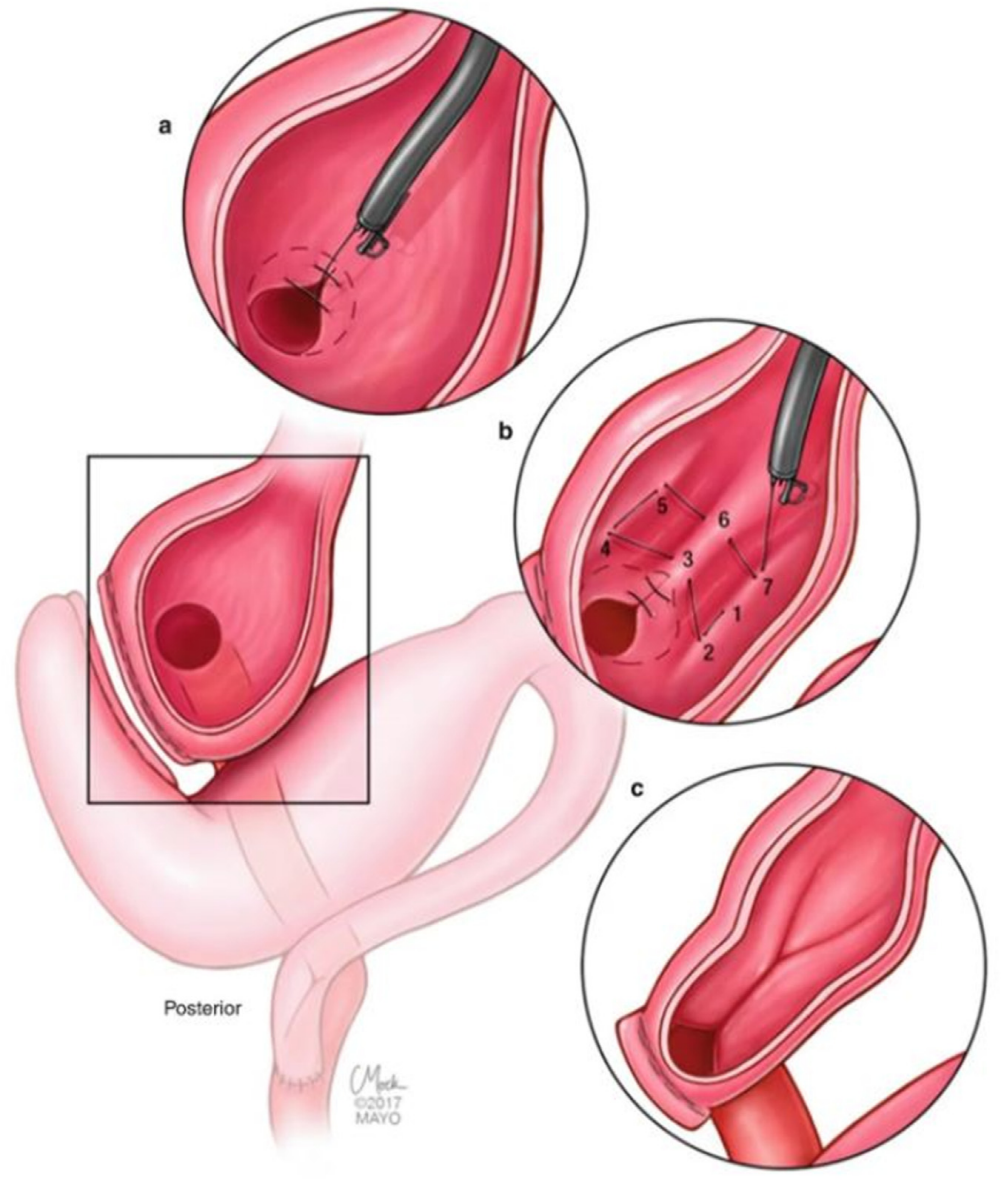
Steps of the enhanced transoral outlet reduction procedure.

**Figure 2:**
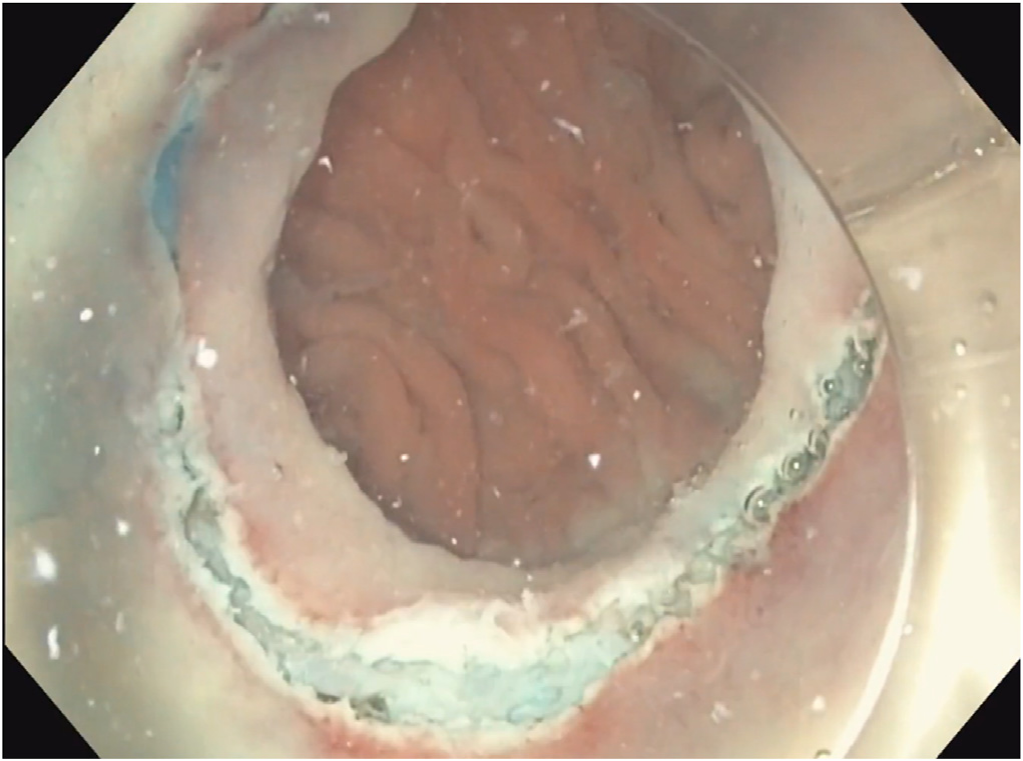
Endoscopic submucosal dissection.

**Figure 3:**
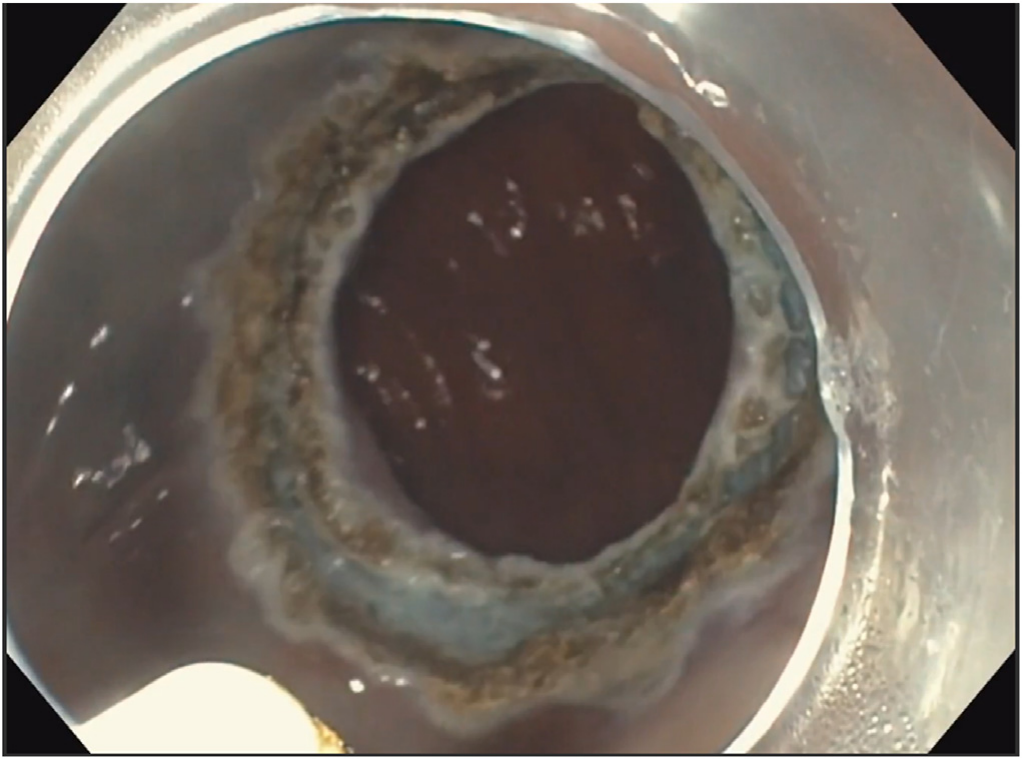
Argon plasma coagulation to expand the submucosal space.

**Figure 4:**
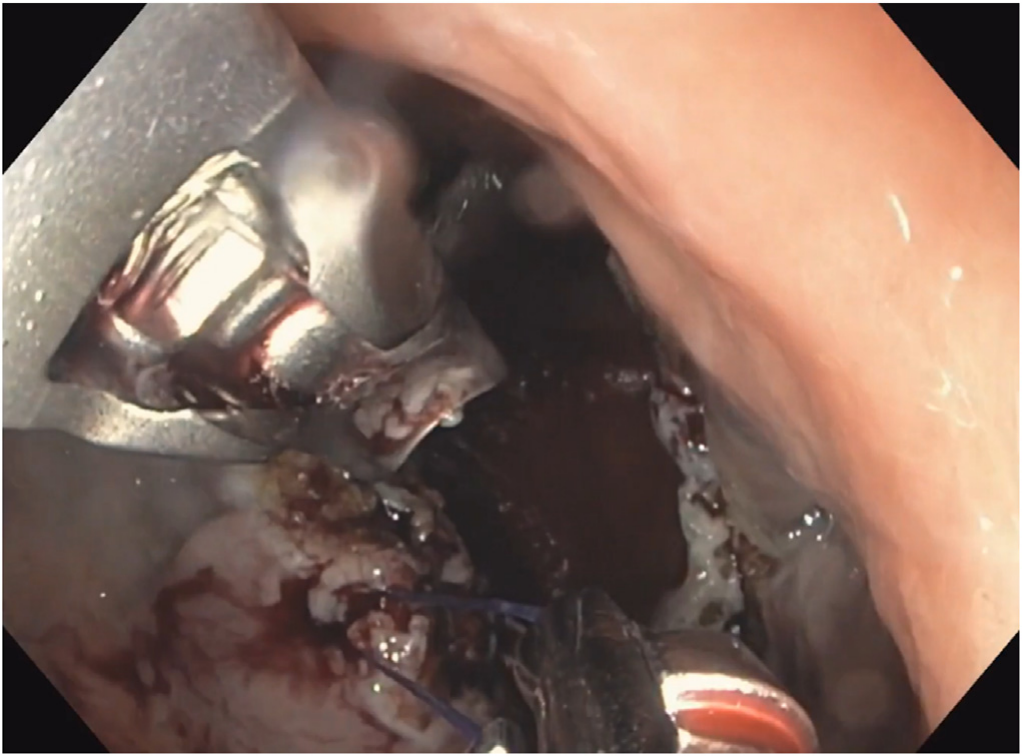
Zipper reduction of the gastrojejunal anastomosis.

**Figure 5:**
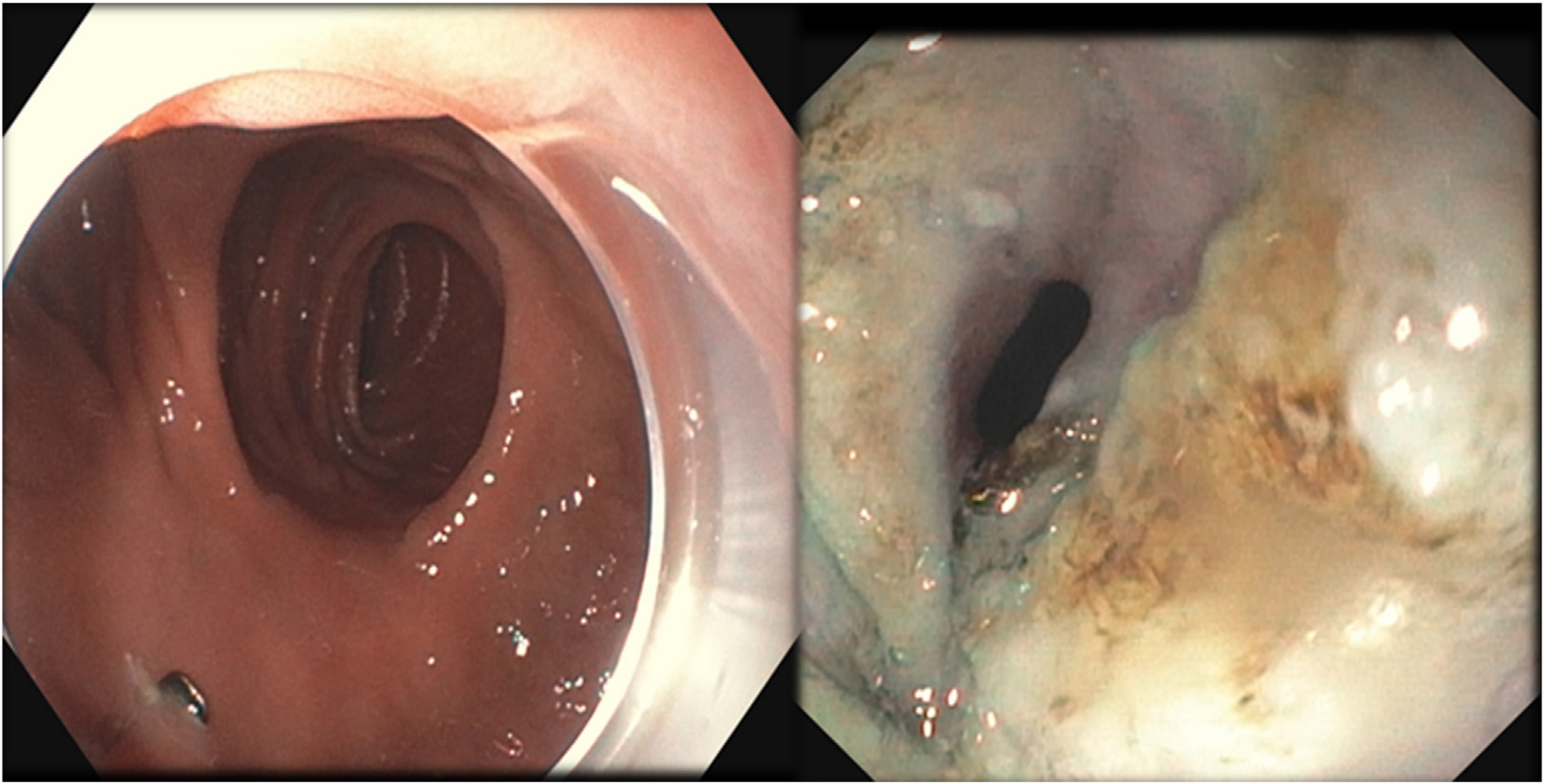
Narrowing of the anastomosis.

**Figure 6:**
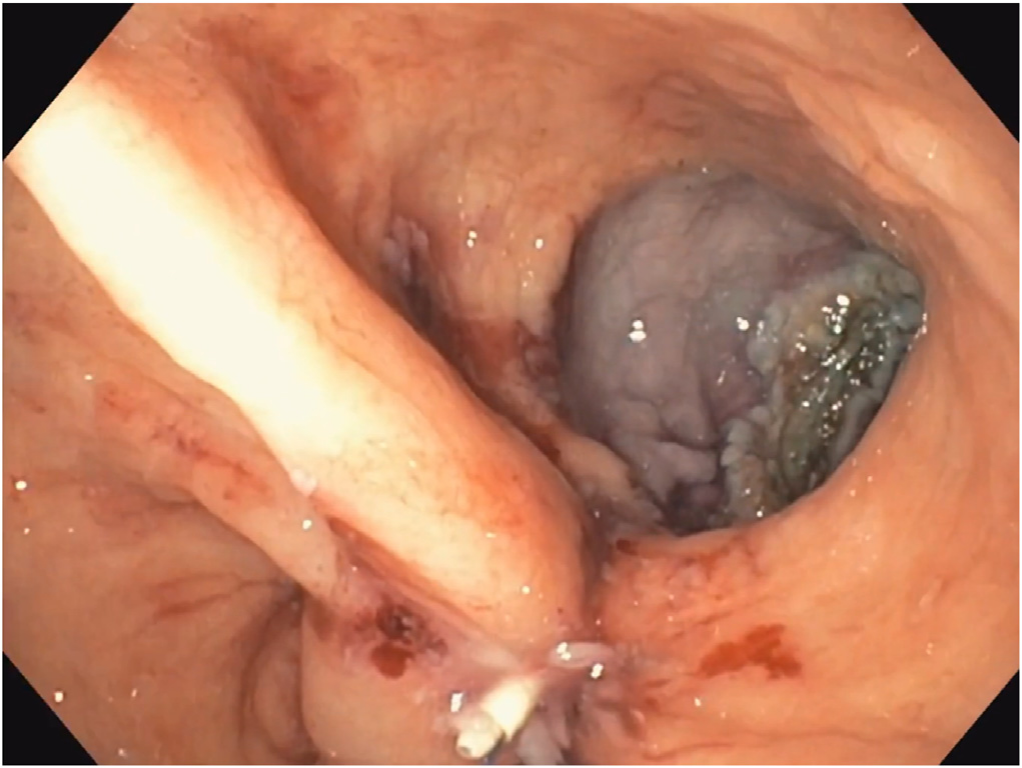
Tuberization of the gastric pouch.

**Figure 7:**
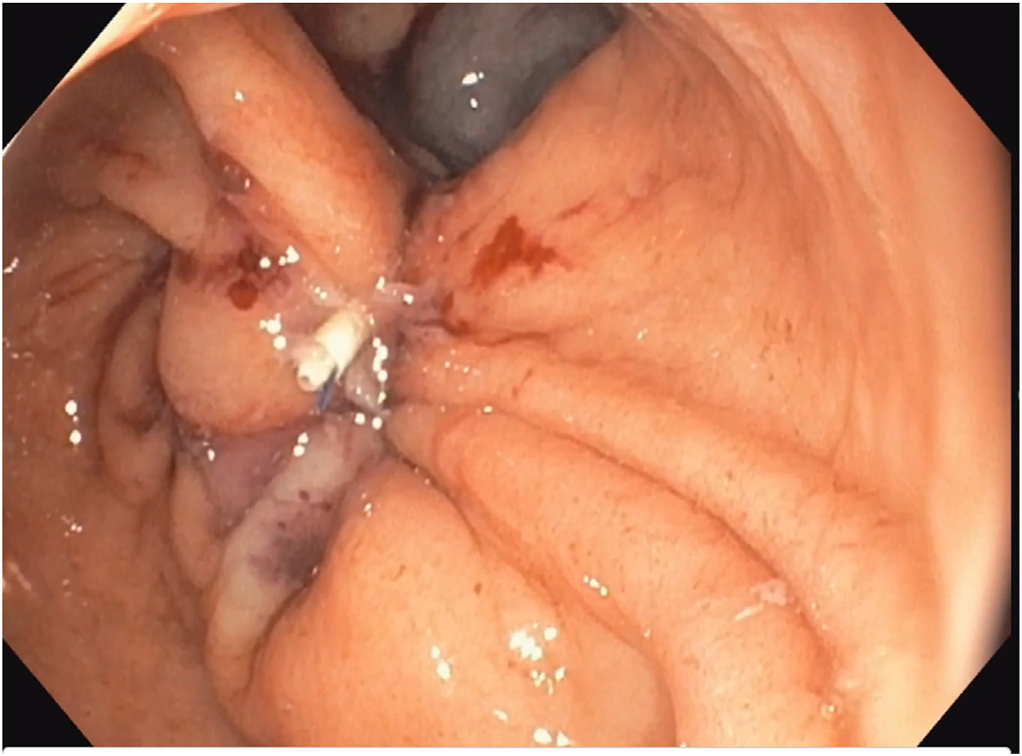
Structured tubular outlet.

A 53-year-old woman presented for weight recurrence after RYGB. She had undergone RYGB in 2010, with an initial weight of 110 kg (body mass index [BMI] 37.6 kg/m^2^). Nadir weight achieved was approximately 78.9 kg (BMI 27 kg/m^2^), which she had maintained up until 1 year before presentation. The patient's weight gradually increased, reaching 107 kg (BMI 36.5 kg/m^2^), and she also began experiencing reflux symptoms. Multiple weight loss attempts, including pharmacotherapy and lifestyle modifications, were unsuccessful. After evaluation by a multidisciplinary team, we proceeded with the enhanced TORe procedure (Fig. 1). The procedure began with an endoscopic submucosal dissection of the GJA, with prophylactic cautery (Fig. 2) of submucosal vessels performed carefully to prevent bleeding. Next, argon plasma coagulation (Fig. 3) was applied to mucosal surfaces, expanding the submucosal space and enhancing visibility. After this, a “zipper-reduction” technique was used to reduce the GJA using endoscopic suturing (Fig. 4), effectively narrowing the anastomotic opening (Fig. 5). Finally, the distal gastric pouch was tubularized (Fig. 6), creating a structured tubular outlet to optimize gastric flow (Fig. 7) (Video 1, available online at at www.videogie.org).

**Figure 8:**
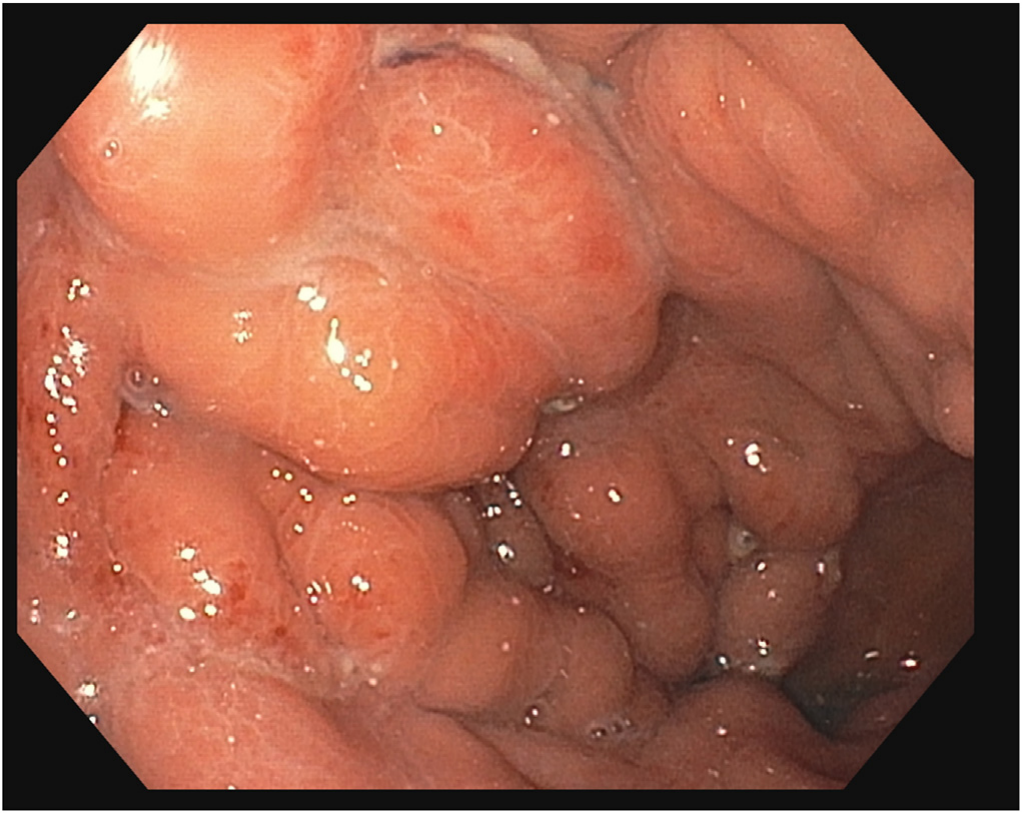
Follow-up EGD at 6 months.

At 6-month follow-up (Fig. 8), the patient reported with a weight of 92 kg, representing 14% total body weight loss with improvement in her GERD symptoms and overall health. No adverse events were observed during that period.

## Conclusions

We used tubular TORe in our reduction of the gastric pouch into a tubular outlet to decrease gastric capacity and motility for a more sustained effect. We also illustrated an uncommon GJA zipper-like suture pattern resulting in efficacious and safe outcomes after TORe. More controlled studies are needed to investigate the efficacy of this pattern compared with more common ones (purse-string, interrupted).

## Patient Consent

The patient in this article has given written informed consent to publication of their case details.

## Disclosure

All authors disclosed no financial relationships.
